# Subanesthetic dose of ketamine for the antidepressant effects and the associated cognitive impairments of electroconvulsive therapy in elderly patients—A randomized, double‐blind, controlled clinical study

**DOI:** 10.1002/brb3.1775

**Published:** 2020-12-11

**Authors:** Lei Zou, Su Min, Qibin Chen, Xiao Li, Li Ren

**Affiliations:** ^1^ Department of Anesthesiology The First Affiliated Hospital of Chongqing Medical University Chongqing China; ^2^ Departments of Psychiatry the First Affiliated Hospital of Chongqing Medical University Chongqing China

**Keywords:** depression, elderly, electroconvulsive therapy, ketamine

## Abstract

**Objectives:**

We previously confirmed that low‐dose ketamine, as an adjunctive anesthetic for electroconvulsive therapy (ECT) in adult patients with depression, accelerates the effects of ECT and reduces the ECT‐induced learning and memory deficits. This study explored the efficacy and safety of low‐dose ketamine in elderly patients with depression.

**Methods:**

Elderly patients with depression (*N* = 157) were randomly divided into two groups: propofol anesthesia group (group P) and propofol combined with ketamine anesthesia group (group KP). Patients in group KP were given low‐dose ketamine (0.3 mg/kg) for each ECT treatment; patients in group P were given the same amount of normal saline. Depressive symptoms and global cognitive functions were assessed using the 24‐item Hamilton Depression Rating Scale and Mini‐Mental State Examination, respectively, at baseline, 1 day after the 1st, 2nd, 4th, and 6th ECT sessions, and 1 day after the end of the ECT course. ECT effects of and complications were recorded.

**Results:**

In total, 67 patients in group KP and 70 in group P completed the study. After the ECT, the response and remission rates were 82.09% and 73.13%, respectively, in group KP, and 81.43% and 68.57%, respectively, in group P; there was no statistical difference between groups. However, the incidence of cognitive function impairment was lower in group KP (10.4%) than in group P (25.7%), while different electrical dose and seizure duration were required during the course of treatment between the two groups. There was no difference in the complications of ECT between groups.

**Conclusions:**

Low‐dose ketamine is safe as an adjunct anesthetic for elderly patients subjected to ECT. It has a protective effect on cognitive function and may accelerate the antidepressant effects of ECT.

## Graphical abstract

This study will explore the efficacy and safety of low‐dose ketamine in elderly patients with depression. Low‐dose ketamine was safe as an assistant anesthetic for elderly patients with ECT. It had a protective effect on cognitive function and may accelerate the effect of ECT.

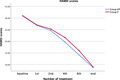

## 1 INTRODUCTION

According to the World Health Organization, depression is the fourth largest disease burden in the world, one of the main causes of functional disability, with high rates of incidence, recurrence, and suicide, as well as high medical costs. By 2020, depression is expected to become the second largest disease after cardiovascular disease worldwide. In China, the incidence of depression is about 6%, while about 30 million people have been diagnosed with depression at present (Huang et al., 2019).

Oral antidepressants are the first choice to treat depression. A large number of clinical studies have confirmed that although their curative effect is accurate, it is limited by the slow onset and low efficiency. Compared with the curative effect of drug therapies, electroconvulsive therapy (ECT) for depression has a higher efficiency rate and quicker onset (Dong et al., 2018; Rhebergen et al., 2015), especially after the introduction of anesthetics and muscle relaxants used during the procedure. The modified ECT not only eliminates the patients' fear and reduces the stress response, but also has been widely used worldwide as it reduces the incidence of adverse events, such as fracture, tooth injury, and cardiovascular and cerebrovascular accidents. However, the possible cognitive impairments caused by ECT are still a complication that needs attention.

It was reported that intravenous injection of small doses of ketamine can improve the cognitive functions of patients with depression (MacPherson & Loo, 2008). Based on this clinical report, our team confirmed that the dose of ketamine in flaxen intoxication reduces learning and memory impairments in a rat depression model after ECT (Hao, Zhu, Li, Lv, & Min, 2016). Furthermore, a previous clinical study confirmed that propofol anesthesia combined with the dose of ketamine reduces the ECT treatment time in adult patients. Specifically, it was shown to reduce learning and memory impairments, improve cognitive impairments induced by ECT, and reduce ECT treatment time, without inducing significant adverse effects (Chen et al., 2017).

With the expansion of the aging population in China, the number of elderly patients with depression is also on the rise. However, in our previous study, we only focused on adult patients (18–65 years old), and thus, we did not examine the effectiveness of ketamine in protecting the cognitive functions of elderly patients subjected to ECT.

Therefore, this study explored a more effective ECT anesthesia regimen for elderly patients, so that it may be ultimately used to reduce the ECT‐induced cognitive impairments and to protect the patients’ cognitive functions after ECT at any age. We included elderly patients with depression, who were administered with ketamine as the auxiliary drug of propofol anesthesia for ECT, and examined whether this drug combination improves ECT efficiency, including ECT’s antidepressant effects, and protects the patients’ cognitive functions.

## 2 MATERIALS AND METHODS

### 2.1 Study design and participants

The patients were enrolled for ECT at the Department of Psychiatry and Anesthesiology of the First Affiliated Hospital of Chongqing Medical University from January 2018 to January 2019. The study protocol was approved by the ethics committee of the hospital (No. 20140409), and written consent was acquired from all subjects. The study was registered at http://www.chictr.org.cn (No. ChiCTR1800015082).

Subjects older than 60 years diagnosed with major depression (Diagnostic and Statistical Manual of Mental Disorders, Fourth Edition) were included. We excluded patients diagnosed with other psychiatric diseases; patients with severe illness; and those with contraindications for ECT and anesthesia, such as severe cardiovascular and cerebrovascular disease, history of allergy for anesthetics, history of seizures, and severe hepatic and renal dysfunction.

Based on a computer‐generated random number table, the recruited subjects were randomly divided into two groups: group KP and group P. Pretest verification before this study showed a 30%–35% incidence of cognitive impairment after ECT in our hospital, decreasing to about 10% after ketamine administration. For a statistical power of 80% at a 0.05 significance level, we determined that 59 subjects would be required in each group; considering a 10% dropout rate, the final sample size was calculated to be at least 132 subjects. The sample size was calculated by two independent proportions power analysis using PASS 11.

### 2.2 Anesthesia

Electrocardiogram, noninvasive blood pressure measurement, and pulse oxygen saturation analysis were conducted for all subjects. Anesthesia was induced before the ECT. Patients in group P received 1.5 mg/kg propofol (Corden Pharma S.p.A, No: X17073B) and 1 mg/kg suxamethonium chloride (Shanghai Xudong Haipu Pharmaceutical Co. LTD, NO: AA151201) intravenously. The same volume saline was injected as the placebo adjunct anesthetic. Patients in group KP received 0.3 mg/kg ketamine before receiving 1.5 mg/kg propofol and 1 mg/kg suxamethonium chloride n. During the treatment, patients were supported by a mask with 100% oxygen, and a short‐acting β‐blocker was used as necessary.

### 2.3 Electroconvulsive therapy

ECT was conducted three times a week using Thymatron DGx ECT system (Somatics LLC, Lake, IL), with the electrode placed on both temporal sides. The first energy for ECT was determined according to the patient's age: energy percent = age × 0.5%. The stimulation energy was adjusted based on the seizure time. The energy was increased by 5% in the subsequent treatment if the seizure time was <25 s. Patients were usually subjected to 8–12 sessions of ECT, and ECT was stopped if the patients achieved the standard of remission, as described below.

### 2.4 Outcome assessment

Depressive symptoms were assessed using the 24‐item Hamilton Depression Rating Scale (HAMD‐24) at baseline, 1 day after 1st, 2nd, 4th, and 6th ECT sessions, and 1 day after the end of the ECT session. Response was defined as a ≥50% decrease in HAMD‐24 scores from baseline. Remission was defined as a <10 in HAMD‐24 scores after two consecutive ECT sessions. In addition, cognitive function was evaluated using the Mini‐Mental State Examination (MMSE) at the above time points. The MMSE comprises 30 items divided into the following components: orientation, registration, attention, delayed recall, language, and praxis. An MMSE score less than 24 is considered to reflect mild cognitive impairment.

The adverse events related to ketamine, such as hallucination and impulsive behavior, were recorded. Additionally, the heart rate, blood pressure, and oxygen saturation during ECT treatment were also recorded. The seizure time was collected from the ECT instrument, and the number of ECT sessions for each patient was recorded.

### 2.5 Statistical analysis

Statistical analyses were conducted using IBM SPSS Statistics 17.0 software (IBM Corporation, Armonk, NY). Continuous variables with normal distributions are presented as mean ± standard deviation, and differences between groups were determined using Student's *t* test. Variance analysis of repeated measurements and the least significant difference *t* test were used for intragroup comparisons. Discontinuous variables were analyzed using chi‐square test. Differences were considered statistically significant when *p* values were <.05.

## 3 RESULTS

### 3.1 Subject characteristics

**Figure 1:**
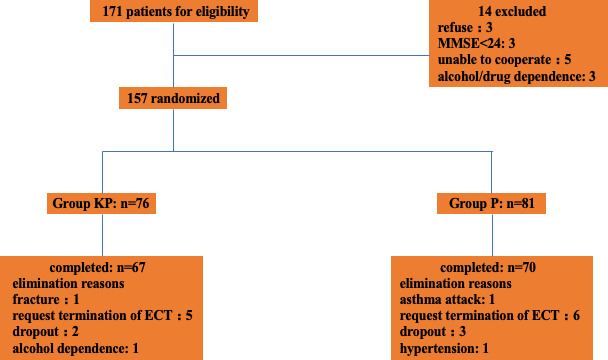
Flowchart demonstrating patient

171 patients were enrolled in this study, and 157 patients were eligible. 76 patients were randomly assigned to group KP, and 81 patients were assigned to group P. The flowchart was shown in Figure 1.

There was no significant difference in demographic characteristics (age, sex, body mass index) between group KP and group P. Baseline HAMD and MMSE scores were also comparable between groups. Moreover, no difference in the use of antidepressants was found between the two groups. Considering the possible influence of risk factors, such as cerebrovascular disease, on depression symptoms and cognitive function, we also analyzed high‐risk factors, such as hypertension, diabetes, atherosclerosis, and hyperlipidemia **(**Table 1).

**Table 1:** Demographic and clinical characteristics

Basic situation	Group KP (*n* = 67)	Group P (*n* = 70)	χ^2^	*p*‐Value
Gender (*n*/percentage)				
Male	24 (35.8%)	23 (32.9%)	–	–
Female	43 (4.2%)	47 (67.1%)	0.034	.853
Age (year)	65.76 ± 3.98	65.62 ± 3.92	–	.834
BMI	22.37 ± 2.35	21.73 ± 2.41	–	.122
Complication (*n*/percentage)				
Hypertension	29 (43.3%)	27 (38.6%)	0.315	.575
Diabetes	9 (13.4%)	15 (21.4%)	1.012	.314
Atherosclerosis	4 (6.0%)	2 (2.9%)	0.223	.637
Hyperlipidemia	6 (9.0%)	8 (11.4%)	0.038	.845
Antidepressant medication (*n*/percentage)				
SSRI	50 (74.6%)	53 (75.7%)	0.003	.960
SNRI	10 (14.9%)	12 (17.1%)	0.015	.904
NASSA	18 (26.9%)	20 (28.6%)	0.001	.974

In group KP, a total of 67 ECT patients were administered, and the average ECT treatment was 9.87 ± 1.48 sessions for each patient. In group P, a total of 70 ECT patients were performed, and the average ECT treatment was 9.99 ± 2.04 sessions for each patient. Compared with group P, the seizures time was higher in group KP in the 1st, 2nd, and 4th ECT sessions, but no significant difference was found in the 6th and the last ECT sessions. Although no difference was found in the electrical dose applied during the 1st and 2nd ECT sessions, significant differences were observed in the 4th, 6th, and the last ECT sessions **(**Table 2).

**Table 2:** Effects of electroconvulsive therapy

	Group KP (*n* = 67)	Group P (*n* = 70)	F	*p*‐Value
Number of treatments	9.87 ± 1.48	9.99 ± 2.04	10.251	.695
Electrical dose (millicoulomb)				
First ECT	168.88 ± 11.62	170.64 ± 10.66	3.418	.356
Second ECT	185.43 ± 17.38	185.04 ± 20.07	0.580	.904
Fourth ECT	202.35 ± 24.41*	215.64 ± 26.32*	2.339	.003
Sixth ECT	225.30 ± 18.55*	232.20 ± 19.14*	0.000	.034
Last ECT	244.48 ± 23.63*	254.16 ± 24.92*	1.478	.021
Seizure duration (second)				
First ECT	49.08 ± 6.32*	46.65 ± 6.68*	0.241	.031
Second ECT	45.91 ± 6.66*	42.83 ± 7.50*	1.176	.012
Fourth ECT	41.06 ± 7.85*	38.26 ± 6.84*	3.032	.027
Sixth ECT	38.10 ± 6.03	36.86 ± 5.76	0.300	.218
Last ECT	32.96 ± 5.43	32.73 ± 4.53	2.093	.791

*p< 0.05.

### 3.2 Antidepressant effects

**Figure 2:**
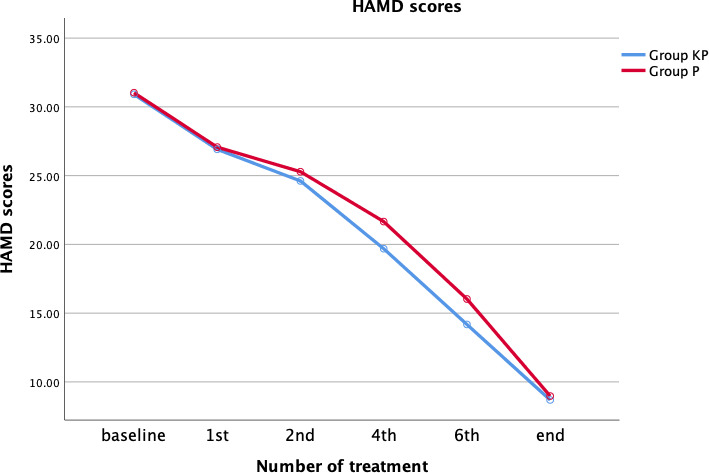
Hamilton depression rating scale score

Repeated measurement analysis of variance was used to determine the influence of the two anesthesia regimens on HAMD scores considering changes in treatment times. Mauchly's test of sphericity did not confirm the hypothesis of sphericity distribution (*p* = .001). There was no statistically significant interaction between treatment time and anesthesia regimen, as assessed by using tests of within‐subjects effects (*F* = 1.471, *p* = .227), and there was no difference in the final effect between the two groups (Figure 2).

HAMD scores in group KP were lower than those in group P after the 4th and 6th sessions of ECT. However, no difference was found after the 1st and 2nd sessions, or at the end of the ECT course. Analysis of the response and remission rates revealed no differences between the two groups at the end of the ECT course; however, after the 6th ECT session, the response rate was higher in group KP **(**Table 3).

**Table 3:** Antidepressant effects of electroconvulsive therapy

	Group KP (*n* = 67)	Group P (*n* = 70)	F	χ^2^	*p*‐Value
HAMD scores					
Baseline	30.91 ± 3.67	31.03 ± 4.11	0.040	–	.860
First ECT	26.91 ± 3.68	27.07 ± 4.17	0.063	–	.811
Second ECT	24.61 ± 3.15	25.29 ± 3.67	1.645	–	.252
Fourth ECT	19.69 ± 3.74*	21.66 ± 3.43*	0.245	–	.002
Sixth ECT	14.18 ± 3.65*	16.03 ± 4.66*	6.439	–	0.011
End of ECT	8.69 ± 4.15	8.97 ± 4.82	2.684	–	0.712
Remission					
End of ECT	49 (73.13%)	48 (68.57%)	–	0.159	.690
Response					
Sixth ECT	51 (76.12%)*	41 (58.57%)*	–	4.270	.039
End of ECT	55 (82.09%)	57(81.43%)	–	0.015	.904

*p< 0.05.

### 3.3 Cognitive impairments

**Figure 3:**
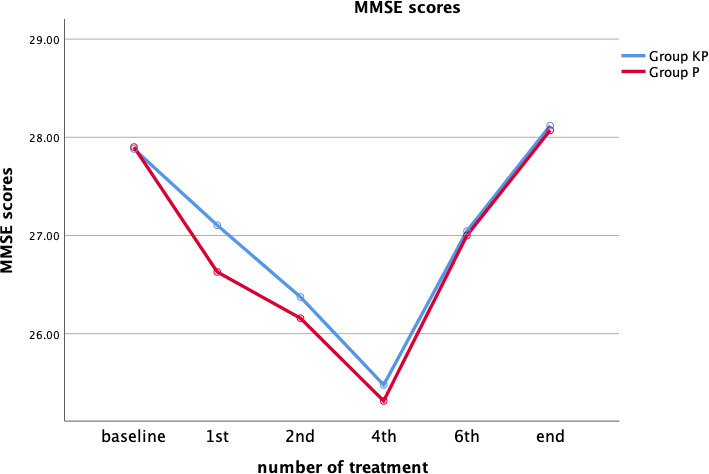
Mini‐mental state examination score

Repeated measurement analysis of variance was used to determine the influence of the two anesthesia regimens on MMSE scores considering the changes in treatment times. Mauchly's test of sphericity did not confirm the hypothesis of sphericity distribution (*p* = .001). There was no statistically significant interaction between treatment times and anesthesia regimen, as assessed by using tests of within‐subjects effects (*F* = 1.043, *p* = .391), and there was no difference in the final effect between the two groups (Figure 3).

Seven patients in group KP and 18 in group P were determined to have cognitive function impairments (MMSE score < 24). The incidence of cognitive function impairment was lower in group KP than in group P. However, no significant differences in MMSE scores were found between the two groups (Table 4).

**Table 4:** Evaluation of cognitive function in two groups

	Group KP (*n* = 67)	Group P (*n* = 70)	*F*	χ^2^	*p*‐Value
MMSE scores					
Baseline	27.88 ± 1.02	27.90 ± 0.98	0.245	–	.910
First ECT	27.10 ± 1.43	26.63 ± 1.38	0.554	–	.050
Second ECT	26.27 ± 1.58	26.16 ± 1.66	0.126	–	.436
Fourth ECT	25.48 ± 1.76	25.31 ± 2.06	1.415	–	.620
Sixth ECT	27.04 ± 1.25	27.00 ± 1.33	0.061	–	.839
End of ECT	28.12 ± 0.96	28.07 ± 0.91	1.254	–	.764
Cognitive impairment	7 (10.4%)*	18 (25.7%)*	–	4.045	.044

*p< 0.05.

### 3.4 Adverse events

Psychiatric and cardiovascular side effects were the main adverse events. There was no serious event of hypertension or tachycardia in the two groups. In group KP, 4 cases of hallucination, 14 cases of myalgia or headache, 7 cases of nausea and vomiting, and 2 cases of delirium were recorded. In group P, 2 cases of hallucination, 16 cases of nausea and vomiting, 5 cases of myalgia or headache, and 2 cases of delirium were reported. No significant differences were found when comparing adverse events between the two groups. Further, the incidence of psychiatric and cardiovascular adverse events was also comparable (Table 5).

**Table 5:** Adverse event in two groups

	Group KP (*n* = 67)	Group P (*n* = 70)	χ^2^	*p*‐Value
Hallucination	4 (6.97%)	2 (2.86%)	0.223	.637
Myalgia or Headache	14 (20.90%)	16 (22.86%)	0.005	.943
Nausea and Vomiting	7 (10.45%)	5 (7.14%)	0.146	.703
Delirium	2 (2.99%)	2 (2.86%)	0.214	.643

## 4 DISCUSSION

The purpose of this study was to investigate the safety of intravenous low‐dose ketamine (0.3 mg/kg) under propofol anesthesia, as well as its efficacy in reducing cognitive impairments caused by ECT. We found no significant psychotropic or hemodynamic adverse effects in patients who received ketamine during ECT. Although the adjunctive low‐dose ketamine did not affect the final response and remission rates, the effects of ECT appeared earlier in the treatment process. Moreover, seizure duration was longer in the early stages of the treatment course and a lower electrical dose was required at the end of the ECT course in patients administered with ketamine plus propofol than in those administered only with propofol. MMSE scores decreased at the beginning of treatment, but there was no difference between the two groups; in addition, there was no difference at the end of the ECT course. However, the incidence of cognitive dysfunction during treatment was higher in patients who received only propofol.

Similarly to most commonly used intravenous anesthetics, propofol has no significant effect on the efficacy of ECT (Bauer et al., 2009). In order to improve this efficacy, several studies have tried to improve various parameters of electric shock treatment in recent years, including the course of treatment, electrical dose, adjunctive anesthesia drugs, and so on (Li et al., 2017; Tzabazis et al., 2017). Ketamine was first used in the clinic as an anesthetic, and many preclinical and clinical studies have shown that it has rapid antidepressant effects(Correia‐Melo, Argolo, Araújo‐de‐Freitas, Leal, & Kapczinski, 2017; Koike, Iijima, & Chaki, 2011; Wilkinson et al., 2018), which last for a long time, even more than one week. Based on its antidepressant effects, researchers have used ketamine as an adjunctive anesthetic for ECT, in order to improve the efficacy of ECT. Since Berman's study, which found that a single subanesthetic (0.5 mg/kg) dose of ketamine elicits an almost immediate and dramatic improvement in patients (Berman et al., 2000), ketamine has been widely accepted for the treatment of depression. Later, it was found that intravenous ketamine improves the cognitive function of patients with depression subjected to ECT(MacPherson & Loo, 2008). However, it was reported that ketamine doses ≥0.5 mg/kg increase diastolic blood pressure during ECT (Zhong et al., 2016). Our team previously found that a smaller dose (0.3 mg/kg) also increases the efficacy of ECT (Chen et al., 2020). Considering that the present study was conducted in elderly patients with hypertension, a smaller dose (0.3 mg/kg) was selected. Studies have shown that ketamine reduces the number of ECT sessions required and shortens the overall course of treatment (Chen et al., 2020). Similar to these studies, we found that, in elderly patients, the combination of low‐dose ketamine with propofol does not enhance the antidepressant effects of ECT and that there is no statistically significant difference in the final response and remission rates between patients administered with this combination or with propofol only. In contrast to these studies, our study suggested that the number of ECT sessions is not reduced in elderly patients, which is similar to the findings by Anderson (Anderson et al., 2017), although the population in that study did not include elderly patients, and the sample size was too small. There are two possible explanations for our results. Firstly, due to the fear of cardiovascular side effects in the elderly, we only used 0.3 mg/kg ketamine, whereas other studies even used 1 mg/kg of ketamine. Secondly, since old age is generally associated with high‐risk factors, such as hypertension, diabetes, atherosclerosis, hyperlipidemia, or other cerebrovascular diseases, these factors could have aggravated the depression symptoms of patients(Oudega et al., 2020; She et al., 2019; Wändell, Carlsson, Sundquist, & Sundquist, 2018), thus increasing the number of required ECTs; therefore, addition of low‐dose ketamine might not have been able to significantly accelerate the antidepressant effects of ECT, without exceeding the ceiling effect. However, we found that, in the early stages of treatment, the seizure duration was longer with the adjunctive low dose of ketamine than with propofol only. Moreover, HAMD scores were also significantly lower in the ketamine plus propofol group than in the control, propofol‐only group, indicating a possible acceleration effect by the low‐dose ketamine in the elderly patients. Future studies are required using higher doses of ketamine to examine these effects, to ensure the patients’ safety.

Cognitive impairment is a complication of ECT. Cognitive function can be assessed by measuring learning and memory abilities. Memory impairment after ECT could be a consequence of indiscriminate activation or saturation of glutamate receptors during treatment, as well as disruption of hippocampal plasticity, which is involved in memory; therefore, ketamine may theoretically combat these effects (Maeng & Zarate Jr, 2007). Our team previously found that a low dose of ketamine (0.3 mg/kg) as an adjunctive to propofol in adults significantly reduces the effects of ECT on a range of aspects relative to memory, using a modified Chinese version of the Wechsler Memory Scale (Chen et al., 2017). In the present study, the MMSE test was used to assess cognitive functioning. The MMSE is a global cognitive screening instrument to assess orientation, attention, executive functioning, and memory (Ciesielska et al., 2016). Although MMSE is believed not to be specific and sensitive enough due to its simplicity and operability, most research teams still use this instrument as a preliminary test to assess the effects of ECT on cognitive function (Kirov, Owen, Ballard, Leighton, & Hannigan, 2016). In our study, cognitive dysfunction was reported in both groups, confirming that ECT may cause cognitive impairment and that MMSE may be used as a preliminary assessment tool of cognitive function after ECT. Moreover, we found that MMSE scores of patients in both groups decreased significantly at the beginning of treatment, although not all patients met the criteria of cognitive dysfunction diagnosis, further suggesting that ECT could cause mixed cognitive impairment in the elderly. Although MMSE scores in both groups returned to baseline values after treatment, this does not suggest that electroshock does not damage cognitive function at the end of the treatment. Instead, the recovery of MMSE scores might reflect the relief of depression symptoms and the improvement of cognitive ability (Obbels, Verwijk, Vansteelandt, Dols, & Bouckaert, 2018; Verwijk et al., 2014). Alternatively, as some reports have pointed out, a learning effect cannot be excluded with the MMSE scale, as it is relatively simple and has been tested many times in patients (Obbels et al., 2019). We also found that the incidence of cognitive dysfunction was still higher in the propofol‐only group than in the ketamine plus propofol group throughout the treatment process, indicating that ketamine protects cognitive function, possibly due to the lower electrical dose finally used during the ECT course.

Further, our study showed that ketamine as an adjunctive anesthetic in elderly patients does not increase the risk for cardiovascular and cerebrovascular diseases, nor the adverse events of ECT. This could be explained by the fact that we used a low dose of ketamine (0.3 mg/kg). Increasing the dose within a certain range may enhance the beneficial effects of ketamine, but may lead to more mental symptoms and cardiovascular adverse events (Ainsworth, Sepehry, & Vila‐Rodriguez, 2019; Ren, Deng, Min, Peng, & Chen, 2018). This is a limitation of our study. Thus, in order to balance efficacy and safety, future studies should examine the effects of different doses of ketamine as an adjunctive anesthetic for ECT in elderly patients.

In conclusion, for elderly patients with depression, adjunctive low‐dose ketamine does not increase the effects of ECT but does increase seizure duration in the early stages of treatment, which may accelerate the onset of ECT effects, and protects against cognitive function impairments due to ECT.

## CONFLICTS OF INTEREST

Authors have no conflicts of interest to report.

## AUTHOR CONTRIBUTIONS

Su Min designed the research experiments; Lei Zou and Xiao Li performed the experiments; Qibin Chen and Li Ren analyzed the data; and Lei Zou wrote the manuscript. All authors discussed the results and reviewed the manuscript.

### Peer Review

The peer review history for this article is available at https://publons.com/publon/10.1002/brb3.1775.

## Data Availability

The data that support the findings of this study are available from the corresponding author upon reasonable request.
